# Xiaoyaosan Improves Depressive-Like Behaviors in Mice through Regulating Apelin-APJ System in Hypothalamus

**DOI:** 10.3390/molecules23051073

**Published:** 2018-05-03

**Authors:** Zhiyi Yan, Haiyan Jiao, Xiufang Ding, Qingyu Ma, Xiaojuan Li, Qiuxia Pan, Tingye Wang, Yajing Hou, Youming Jiang, Yueyun Liu, Jiaxu Chen

**Affiliations:** 1 School of Traditional Chinese Medicine, Beijing University of Chinese Medicine, Beijing 100029, China; 15010190928@163.com (Z.Y.); jiao.hy@foxmail.com (H.J.); d184728208@gmail.com (X.D.); 15652608965@163.com (X.L.); pqx1126@sina.com (Q.P.); Wty1307@163.com (T.W.); yajingHou@163.com (Y.H.); castenyy@gmail.com (Y.J.); chloelou@126.com (Y.L.); 2 Formula-pattern Research Center, School of Traditional Chinese Medicine, Jinan University, Guangzhou 510632, Guangdong, China; 20140941026@bucm.edu.cn

**Keywords:** Xiaoyaosan, depression, apelin, APJ, chronic unpredictable mild stress, hypothalamus

## Abstract

*Background:* The apelin-APJ system has been considered to play a crucial role in HPA axis function, and how the traditional Chinese compound prescription Xiaoyaosan regulates the apelin-APJ system as a supplement to treat depressive disorders. *Objective:* To investigate the depression-like behaviors and expression of apelin and APJ in hypothalamus of chronic unpredictable mild stress (CUMS) mice and study whether these changes related to the regulation of Xiaoyaosan. *Methods:* 60 adult C57BL/6J mice were randomly divided into four groups, including control group, CUMS group, Xiaoyaosan treatment group and fluoxetine treatment group. Mice in the control group and CUMS group received 0.5 mL physiological saline once a day by intragastric administration. Mice in two treatment groups received Xiaoyaosan (0.25 g/kg/d) and fluoxetine (2.6 mg/kg/d), respectively. After 21 days of modeling with CUMS, the expression of apelin and APJ in hypothalamus were measured by real-time fluorescence quantitative PCR, western blot and immunohistochemical staining. The physical condition, body weight, food intake and behavior tests such as open field test, sucrose preference test and force swimming test were measured to evaluate depressive-like behaviors. *Results:* In this study, significant behavioral changes were found in CUMS-induced mice, meanwhile the expressions of apelin and APJ in the hypothalamus were changed after modeling. The body weight, food-intake and depressive-like behaviors in CUMS-induced mice could be improved by Xiaoyaosan treatment which is similar with the efficacy of fluoxetine, while the expressions of apelin and APJ in hypothalamus were modified by Xiaoyaosan. *Conclusions:* The data suggest that apelin-APJ system changes in the hypothalamus may be a target of depressive disorders, and the beneficial effects of Chinese compound prescription Xiaoyaosan on depressive-like behaviors may be mediated by the apelin-APJ system.

## 1. Introduction

Depression is a common mental health problem in modern society, and one of its important inducing factors is the stress response, which is defined as an uncoordinated state of organism or the instability of the internal environment [1]. The main characteristic of stress response is the hyperactivity of the hypothalamus-pituitary-adrenal (HPA) axis and autonomic nervous system after stimulation by the internal and external environment [2]. The HPA axis, as a neuroendocrine immune network hub, has the responsibility to maintain a stable internal environment and provide physiological and psychological responses to the external environment and emotional stimulation [3]. Therefore, hyperactivity of HPA axis is one of the most common neurological manifestations of depression. Corticotropin-releasing factor (CRF) is a key factor in regulating the stress response, and the hypersecretion of CRF in the hypothalamus is the basis of HPA axis dysfunction [4]. The secretion of CRF in the hypothalamus increased when a subject is exposed to stressors, then the secretion of adrenocorticotropic hormone (ACTH) in the pituitary increases, which leads to the increase of glucocorticoid (GC) secreted by the adrenal gland [5]. The main sites of action of GC in the brain are the hippocampus and cerebral cortex, and excess GC can damage the brain by affecting the glucocorticoid receptor (GR) [6], while the excess of GC causes a negative feedback regulation in the dysfunction of the HPA axis, which leads to hyperactivity of the HPA axis [7].

Putative apelin receptor (APJ) related to the angiotensin receptor AT1, is isolated from the human genome in 1993 for the first time [8]. The APJ endogenous ligand (apelin), as a kind of bioactive peptide, has extensive physiological functions through the interaction with its specific receptor APJ [9]. The apelin-APJ system is involved in the functional activities of many systems such as the cardiovascular system, central nervous system, endocrine system, immune system, digestive system and circulatory system [10,11,12,13]. APJ and apelin are distributed in the hypothalamic nuclei [14], especially in the supraoptic nucleus (SON) and paraventricular nucleus (PVN) [15]. In O’Carroll et al.’s study, stress could decrease the expression of apelin in the PVN and the content of cortisol in plasma, and increase the expression of APJ which is associated with the expression of apelin and cortisol (CORT), which means that the apelin-APJ system plays an important role in the generation of GC and negative feedback of the HPA axis [16]. Apelin-13 can promote the release of secretion of ACTH and CORT by stimulating the release of CRF and AVP in the hypothalamus, which suggests that the regulation of apelin depends on CRF and AVP [17]. Furthermore, the central injection of (Pyr1) apelin-13 can increase the expression of c-fos gene in PVN [18], and apelin could significantly stimulate the release of CRF and AVP in hypothalamic explants in vitro [19]. These studies indicate that the apelin-APJ system plays a role in regulating the activity of the HPA axis.

Xiaoyaosan is a classic Chinese compound prescription that has the effect of invigorating the spleen, dispersing the stagnated liver-energy and nourishing blood [20]. It has been widely used to treat mental disorders, particularly depression [21,22]. Modern studies have indicated that Xiaoyaosan has an anti-depressant effect through regulation of the HPA axis [23]. The apelin-APJ system is involved in the disorders of HPA axis caused by depression, therefore, the purpose of the this study is to characterize the expression of apelin and APJ in hypothalamus of mice with chronic unpredictable mild stress (CUMS), and observe the effect of Xiaoyaosan intervention on the apelin-APJ system to determine the anti-depressant mechanism of action of Xiaoyaosan.

## 2. Results

### 2.1. Effect of Xiaoyaosan on Physical Condition, Body Weight and Food Intake of CUMS-Induced Mice

**Figure 1:**
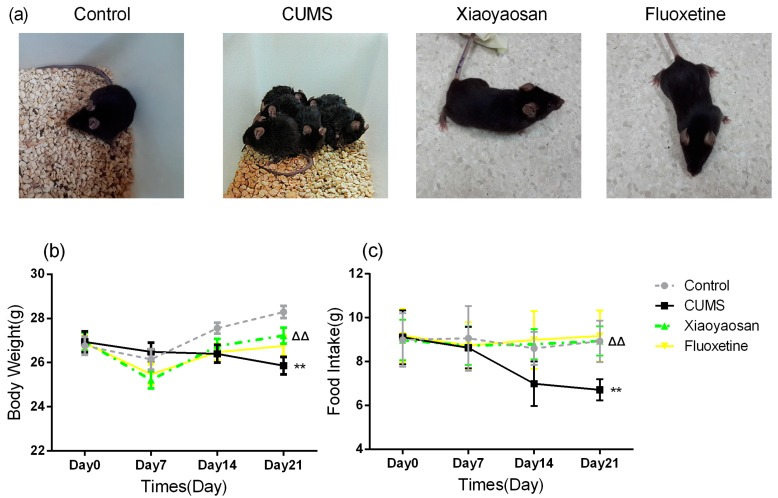
(**a**) Physical condition of mice in all groups was observed at the end of modeling; (**b**) Body weight was recorded once a week during the modeling period; (**c**) Food intake was recorded once a week during the modeling period. Data were expressed as means ± SEM (*n* = 15), ** *p* < 0.01 versus control group; **^ΔΔ^** *p* < 0.01 versus CUMS group.

The observations to characterize the effects of Xiaoyaosan included the physical condition, food intake and body weight, and were carried out in mice with the aim of evaluating the efficacy of Xiaoyaosan in CUMS-induced mice. After modeling for 21 days, compared with the control group the mice in the CUMS group exhibited signs of fatigue, disheveled hair, less activity, flocking together behavior and loose stools, while the physical condition of the two treatment groups was better than that of the CUMS group (Figure 1a). As shown in Figure 1b,c, body weight and food intake in each group showed no significant differences at day 0, and the body weight of mice in all groups also had no significant differences at day 0 and day 7 (*p* > 0.05, respectively), the data of day 14 showed that the mice in the CUMS group had a significant decrease in body weight compared with control group mice (*p* < 0.05), then the difference of body weight between CUMS group and control group became more obvious by day 21 (*p* < 0.01), furthermore, the body weights of mice in the Xiaoyaosan treatment group were higher than those of the CUMS group mice (*p* < 0.05), while the body weight of mice in the fluoxetine treatment group had no significant difference compared with CUMS group mice (*p* > 0.05). The data of food intake showed that the food intake of mice in CUMS group decreased at day14 and day 21 compared with control group (*p* < 0.01), meanwhile, the food intake of Xiaoyaosan and fluoxetine treatment groups at day 14 and day 21 were higher than those of CUMS group mice (*p* < 0.01).

### 2.2. Effect of Xiaoyaosan on Depressive-Like Behaviors of CUMS-Induced Mice

**Figure 2:**
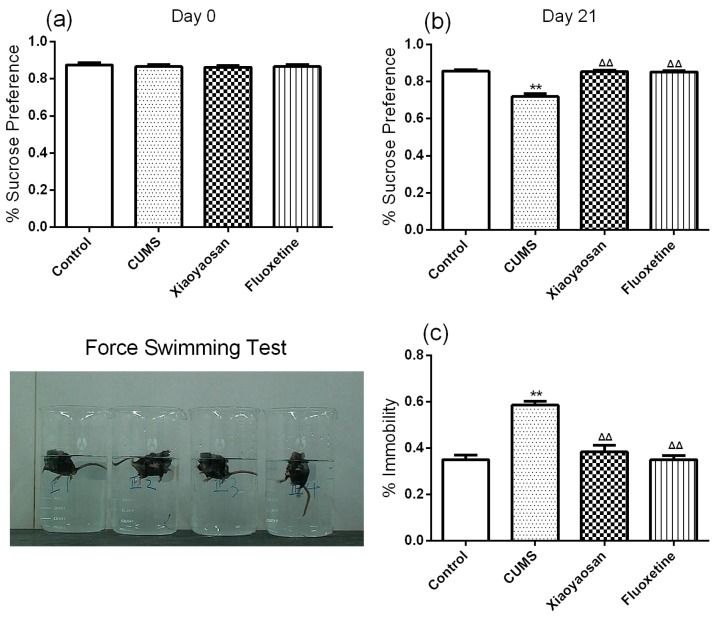
(**a**) SPT of mice in each group at day 0 (baseline); (**b**) SPT of mice in each group at day 21; (**c**) FST of mice in each group at day 21. Data were expressed as means ± SEM (*n* = 15), ** *p* < 0.01 versus control group; **^ΔΔ^** *p* < 0.01 versus CUMS group.

**Figure 3:**
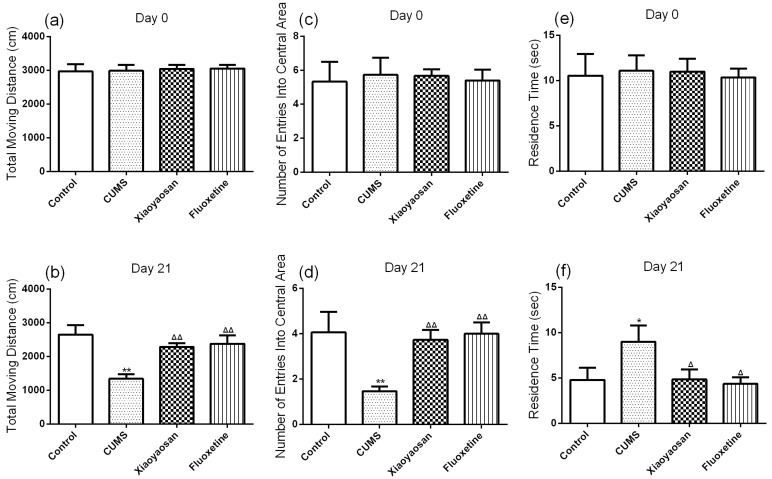
(**a**) The total moving distance of mice in each group at day 0 (baseline); (**b**) The total moving distance of mice in each group at day 21; (**c**) The number of entries into central area of mice in each group at day 0 (baseline); (**d**) The number of entries into central area of mice in each group at day 21; (**e**) The residence time of mice in each group at day 0 (baseline); (**f**) The residence time of mice in each group at day 21. Data were expressed as means ± SEM (*n* = 15), * *p* < 0.05, ** *p* < 0.01 versus control group; **^Δ^** *p* < 0.05, **^ΔΔ^** *p* < 0.01 versus CUMS group.

For the purpose of further evaluating the depressive-like behaviors, several behavioral tests were carried out, including sucrose preference test (SPT), force swimming test (FST) and open field test (OFT). The sucrose preference test is a classic method for detecting loss of pleasure due to depression, and the reduced preference for sucrose in the test is a key indicator of depression in rodents [24]. The SPT results of each group were basically the same at day 0 (both *p* > 0.05), but the sucrose preference of mice in the CUMS group was significantly lower compared with mice in the control group at day 21 (*p* < 0.01), meanwhile the sucrose preference of mice in the two treatment group was significantly higher than those of the CUMS group mice (*p* < 0.01). The forced swimming test is a behavioral despair test to evaluate the efficacy of antidepressant drugs, where the immobility time of mice in the CUMS group was significantly longer compared with control group mice at day 21 (*p* < 0.01, Figure 2c). Similar to the SPT results, the immobility time of the two treatment group mice was significantly lower than those of CUMS group mice (both *p* < 0.01) indicating that both Xiaoyaosan and fluoxetine had antidepressant effects in the CUMS model mice. The open field test reflects the independent and exploratory behaviors of mice in a strange environment [25]. Among all the groups, there was no significant difference in the results of OFT at day 0 (both *p* > 0.05, Figure 3a,c,e). After modeling for 21 days, the total movement distance and the number of entries into the central area of mice in the CUMS group were significantly less than those of control group mice (both *p* < 0.01, Figure 3b,d), while the Xiaoyaosan and fluoxetine treatments remarkably reversed the CUMS-induced decrease in the total movement distance and number of entries into the central area (both *p* < 0.01). 

**Figure 4:**
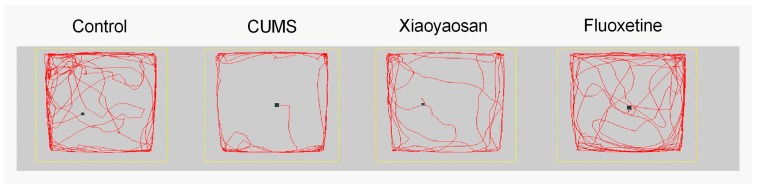
The movement trails of mice in each group at day 21 assessed by video tracking software in the OFT that displayed the locomotor function.

The residence time of mice in the CUMS group was significantly longer than in control group mice (*p* < 0.05, Figure 3f), while Xiaoyaosan and fluoxetine could also reverse this CUMS-induced behavior change (*p* < 0.05). Representative movement trails of mice in each group in the OPT at day 21 are shown in Figure 4.

### 2.3. Effect of Xiaoyaosan on the Expression of Apelin and APJ in Hypothalamus of CUMS-Induced Mice

**Figure 5:**
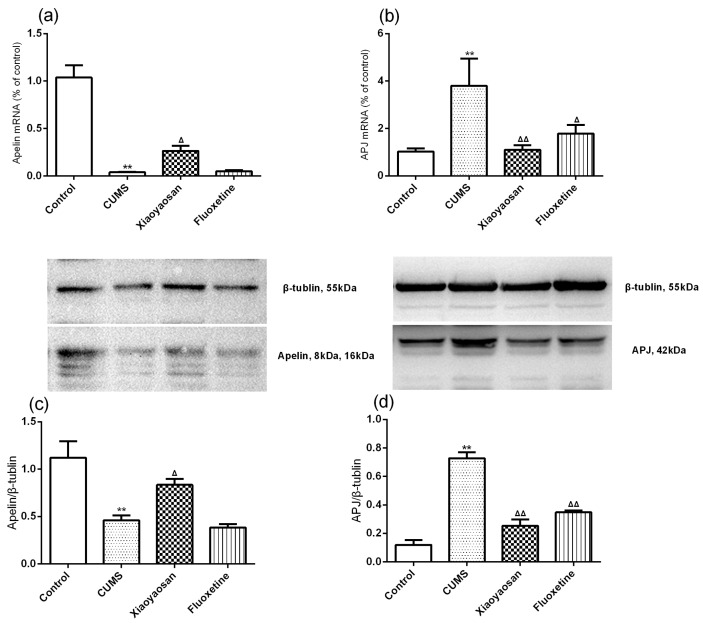
(**a**) The mRNA expressions of apelin in each group; (**b**) The mRNA expressions of APJ in each group; (**c**) The protein level of apelin in each group; (**d**) The protein level of APJ in each group. Data were expressed as means ± SEM (*n* = 5), ** *p* < 0.01 versus control group; **^Δ^** *p* < 0.05, **^ΔΔ^** *p* < 0.01 versus CUMS group.

In order to determine whether Xiaoyaosan regulated the apelin-APJ system in the mouse model of depression, the expressions of apelin and APJ were measured. First of all, the qRT-PCR and western blot results revealed that the 21-day CUMS could significant decrease the apelin level in the hypothalamus of CUMS mice (both *p* < 0.01, Figure 5a,c), and the mice in the Xiaoyaosan treatment group showed a significant increase in apelin level compared with the CUMS group mice (*p* < 0.01). The 21-day CUMS could also significantly increase the APJ level in the hypothalamus of CUMS mice (both *p* < 0.01, Figure 5b,d), and the mice in the Xiaoyaosan treatment group showed a significant decrease in APJ level compared with the CUMS group mice (*p* < 0.01), and a similar result was also observed in the fluoxetine treatment group (*p* < 0.05, *p* < 0.01, respectively). 

**Figure 6:**
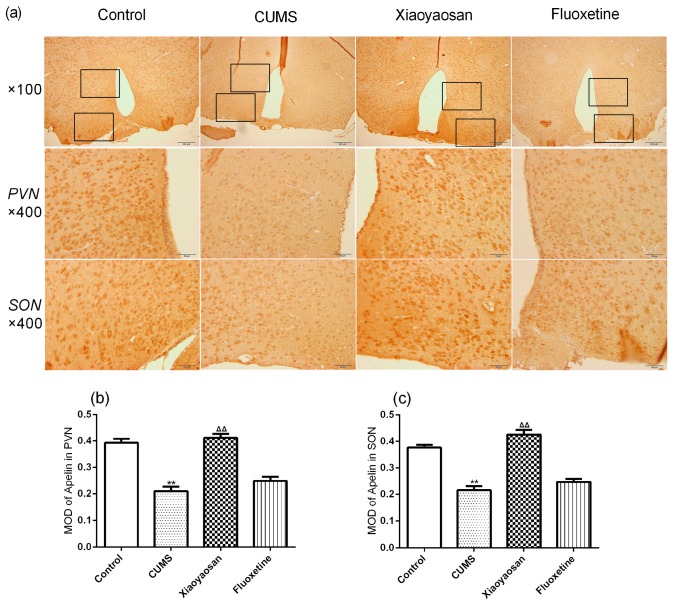
(**a**) Representative micrographs of immunohistochemical staining for apelin (scale bar = 50 μm, 400× magnification) in the PVN and SON; (**b**) The result of image analysis of apelin in PVN; (**c**) The result of image analysis of apelin in SON. MOD, mean optical density. Data were expressed as means ± SEM (*n* = 5), ** *p* < 0.01 versus control group; **^ΔΔ^** *p* < 0.01 versus CUMS group.

**Figure 7:**
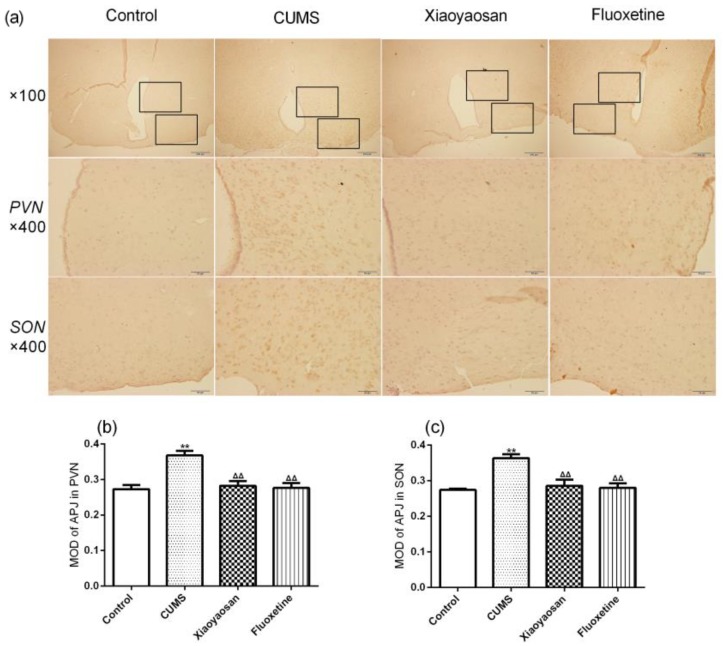
(**a**) Representative micrographs of immunohistochemical staining for APJ (scale bar = 50 μm, 400× magnification) in the PVN and SON; (**b**) The result of image analysis of APJ in PVN; (**c**) The result of image analysis of APJ in SON. MOD, mean optical density. Data were expressed as means ± SEM (*n* = 5), ** *p* < 0.01 versus control group; **^ΔΔ^** *p* < 0.01 versus CUMS group.

The immunohistochemical staining result further indicated that the mice in the CUMS group had a lower expression of apelin and a higher expression of APJ in the PVN and SON compared with the control group (both *p* < 0.01, Figure 6 and Figure 7), while Xiaoyaosan reversed the CUMS-induced changes of apelin and APJ in the PVN and SON (both *p* < 0.01), and the expression of APJ in the PVN and SON of mice in fluoxetine treatment group was also lower than the CUMS group mice (both *p* < 0.01).

## 3. Discussion

CUMS is a common method to establish a stress diathesis model of depression in modern research, and the fact that three weeks of modeling can develop significant depressive-like changes has been widely certified [26,27]. Meanwhile, the C57BL/6J mouse has a homogeneous response to stress, so it is often used to study stress-induced changes in the body [28,29]. Therefore, this study established a mice depression model by three weeks of CUMS, and observed the effect of Xiaoyaosan intervention on the depressive-like behaviors and the changes of the apelin-system in the hypothalamus.

There always exists weight loss and appetite decrease during the depression process [30]. After modeling for three weeks, the mice in the two treatment groups had a better food intake condition than the CUMS group, which suggested that Xiaoyaosan and fluoxetine had similar effects on appetite regulation. A previous study showed that the use of fluoxetine might result in weight loss, although it also has also been reported to have the opposite effects of weight gain and hyperphagia [31,32], so the effects of fluoxetine on weight may be mediated by partially distinct mechanisms and involve some metabolic effects [33,34]. Meanwhile, Xiaoyaosan had a good effect in the regulation of body weight [35], the same results were revealed in this study. The SPT, FST and OFT were used to evaluate the depressive-like behaviors of CUMS-induced mice. The SPT could reflect the stress-induced mental state and estimate the anti-depressant effect of drugs [36]. The SPT results indicated that three weeks of CUMS had an obvious influence on the taste changes of mice, the Xiaoyaosan and fluoxetine treatments could significantly reverse the CUMS-induced anhedonia. The FST in the animal models of depression had good sensitivity and specificity for antidepressant treatments [37]. It had a similar results as SPT in this study that further confirmed the efficacy of Xiaoyaosan and fluoxetine. The OFT, as an animal psychological test, is commonly used to assay the locomotor function and emotionality of rodents [38]. It has the characteristics of simple operation, good feasibility and accurate data records. The total movement distance, number of entries into the central area and residence time were selected to assess the different behaviors of mice in each group. When mice were in a state of fatigue and depression, their locomotor activity would decrease accordingly, so the total movement distance of mice in the CUMS group was significantly lower than that in the control group. The number of entries into the central area reflect the anxiety and adaptability of mice to the new environment, and anxious or depressed mice prefer to move around the box rather than moving into the central area, thus the number of entries into central area of mice in CUMS group was significantly lower than that in the control group. In addition, the residence time represents the retention time of mice to explore the surrounding environment, the curiosity of mice to the strange environment decreased with the increase of residence time, hence an obvious increase could be seen in the residence time of mice in the CUMS group from the data. According to the results discussed in previous studies [21], our study also showed the obvious antidepressant effect of Xiaoyaosan or fluoxetine according to the results of OFT, SPT and FST, furthermore, anxiety-like behaviors, such as significant decrease in number of entries into central area of the OFT could also be improved by the treatment. 

The HPA axis is a vital stress response site, and is also an important regulatory pathway of endocrine activity, HPA axis hyperactivity happens when damage occurs to the negative feedback system of the HPA axis, and then the neuroendocrine immune network becomes imbalanced which is one of the main manifestations of depression [39,40], so the hypothalamus, as the essential center of the HPA axis, is the key region to study the central mechanism of depressive-like behaviors. It is reported that the structures of apelin and APJ are similar in many species such as humans, rats, mice and cows, and the distribution of apelin and APJ has a high degree of overlap in the body [41,42,43]. As a pair of biological active peptides, the role of apelin and APJ in the function of hypothalamus has been explored in many studies [44,45,46], and the changes of the apelin-APJ system in the stress-induced HPA axis dysfunction are also a study point in the mental disorders [47]. To figure out whether apelin-APJ system is involved in the functional mechanism of Xiaoyaosan, the levels of apelin and APJ in the hypothalamus were observed. It was found that CUMS could inhibit the apelin level and increase the expression of APJ in the hypothalamus, which meant that stress might lead to the reduction of apelin, then affect its interaction with APJ, then weaken the function of the apelin-APJ system, and the reason for these changes may be related to compensated agonist loss by increasing the receptor. The same results were reflected in both PVN and SON, which was a supplement to previous research [16]. The results indicated that Xiaoyaosan could promote the apelin-APJ system which was manifested in the significant up-regulation of apelin levels and the normal range of APJ expression. Besides, some research showed that Xiaoyaosan could regulate the content of CRF [48], and the function of the apelin-APJ system was associated with CRF [49], which suggests that the regulating effect of Xiaoyaosan to the apelin-APJ system might be mediated by CRF, but certainly further research is necessary. Fluoxetine is a kind of selective serotonin reuptake inhibitor (SSRI) that is widely used in the treatment of mental diseases such as depression, panic disorders, obsessive-compulsive disorders and bulimia nervosa [50]. The 5-HT system is associated with the HPA axis, and brain 5-HT can enhance the activity of HPA, thus activating the secretion of CRF in the hypothalamus, then promote the secretion of ACTH [51], so fluoxetine was used to set up a reference drug group in this study to evaluate the influence of Xiaoyaosan on CUMS-induced disorder of the hypothalamus. In our study, the expression of apelin in the fluoxetine treatment group had no obvious change compared with the CUMS group, which illustrated that different from Xiaoyaosan, the treatment of fluoxetine might not regulate the apelin level in the hypothalamus of CUMS-induced mice. Further studies are needed to examine the role of Xiaoyosan in the regulation of the apelin-APJ system, and more relative brain regions including cortex, hippocampus and amygdala still needed to be examined, in order to comprehensively analyse the performance of the apelin-APJ system in mental disorders. 

## 4. Materials and Methods 

### 4.1. Animals

Specific-pathogen free (SPF) male C57BL/6J mice (SYXK (Jing) 2012-0001) aged 12 weeks were purchased from Beijing Vital River Laboratory Animal Technology Limited Company (Beijing, China). All mice were adaptive fed for 7 days separately in a standard animal feeding room before the experiments commenced (room temperature: 21 ± 1 °C; relative humidity: 30–40%; light condition: a 12 h/12 h dark/light cycle). All animal experiments were approved by the Institutional Animal Care and Use Committee at Beijing University of Chinese Medicine and conformed to the existing current animal welfare guidelines. The experimental protocols applied in this study were performed in accordance with approved guidelines.

### 4.2. Preparation of Drugs

The compound prescription used in the experiment is Xiaoyaosan which contains the following eight traditional Chinese drugs: Radix Angelicae Sinensis, Radix Paeoniae Alba, Poria, Radix Bupleuri, Radix Glycyrrhizae, Rhizoma Atractylodis Macrocephalae, Herba Menthae and Rhizoma Zingiberis Recens, in a ratio of 6:6:6:6:3:6:2:2. These medicinal herbs were purchased from the Medicinal Materials Company of Beijing Tongrentang (Beijing, China) and extracted in the Chinese medicine preparation room of China-Japan Friendship Hospital as described previously. The extraction rate was 18.8% [52]. 

### 4.3. CUMS Procedure and Drug Administration

A total of 60 mice were randomly divided into four groups (*n* = 15) according to their body weights: control group (no stress with physiological saline), CUMS group (CUMS plus physiological saline), Xiaoyaosan treatment (CUMS plus Xiaoyaosan) group and fluoxetine treatment group (CUMS plus fluoxetine). All mice were housed separately in cages, and the stress assay methods used were restraint stress (3 h), food deprivation (24 h), water deprivation (24 h), high temperature stress (50 °C, 5 min), ice-cold swimming (5 min), unpredictable shocks (50 mV, one shock/10 s, 30 s duration, 15 times in total), tail clamp (1 min), noise environment (60 Hz, 1 h) and wet and soiled cage (24 h), each stress method was used for 2 or 3 times, the CUMS modeling lasted for 21 days. Mice in the control group and CUMS group received 0.5 mL physiological saline by intragastric administration, mice in the two treatment groups received Xiaoyaosan (0.25 g/kg/d) and fluoxetine (2.6 mg/kg/d), respectively [53]. Furthermore, to observe the physical condition of the mice during the CUMS procedure, the body weight and food intake of each mouse were recorded weekly (day 0, day 7, day 14 and day 21) until the end of modeling, the data of the day before experiment (day 0) was recorded as baseline.

### 4.4. Sucrose Preference Test

The sucrose preference test was performed at day 0 and day 21 as previously described [53]. After water and food deprivation 24 h, each mouse was given two bottles containing deionized water and 1% sucrose solution. The ratio of the amount of sucrose solution to that of total solution consumed within 1 h was calculated which represented the parameter of hedonic behavior.

### 4.5. Forced Swimming Test

The forced swimming test was performed at day 21 as previously described [54], mice were individually placed in a clear glass aquarium (19 cm in diameter × 24 cm high) containing 6-cm deep water (24 ± 1 °C) for 6 min. The whole time was recorded using a HD camera, and the immobility time was recorded during the final 5 min. The mouse was considered to be immobile when it stopped swimming and remained floating on the water. The water in the aquarium was changed after each mouse to avoid olfactory cues left by the previous mice.

### 4.6. Open Field Test

The open field test was performed at day 0 and day 21 as previously described [55]. The activity of mice in each group were measured in a 40 cm × 40 cm × 15 cm wooden box without ceiling, the inside wall and floor of which were covered by black paint. The floor was divided into 25 equal squares by white lines, each mouse was placed right in the center and started to record the movement condition for 5 min by HD camera. The box was cleaned after each mouse to avoid olfactory cues left by the previous mice. The Etho Vision 3.0 sofware was used to evaluate the total moving distance, residence time and number of entries into central area.

### 4.7. Sample Collection and Preparation

After modeling for 21 days, the mice in each group were anesthetized by intraperitoneal injection of 3% sodium pentobarbital. The hypothalamus of five mice in each group were collected in frozen pipes and stored at −80 °C for western blot testing, then the hypothalamus of five mice in each group were collected in frozen pipes with RNA preservation solution for qRT-PCR, the whole brains of the remaining mice after fixation of arterial perfusion were stored in 4% paraformaldehyde solution at 4 °C for tissue slicing and immunohistochemistry.

### 4.8. Quantitative Real-Time Polymerase Chain Reaction (qRT-PCR) Analysis

Total RNA of hypothalamus from each mouse was extracted using Trizol reagent (Applied Biosystems, Waltham, MA, USA), and then the RevertAid First Strand cDNA Synthesis Kit (Termo Fisher Scientifc, Waltham, MA, USA) was used to synthesize cDNA. The sequences for primers showed in Table 1 were designed based on published mRNA sequences in NCBI, and then synthesized by a professional biotechnology company (Sangon Biotech Co., Ltd., Shanghai, China). GAPDH (BBI Life Science, Amherst, MA, USA) was used as house-keeping gene in the study. The qRT-PCR reaction system was prepared by SYBR^®^ Green PCR Master Mix (Applied Biosystems) in a final volume of 50 μL and performed on Multicolor Real-time PCR Detection System (Bio-Rad, Hercules, CA, USA). The thermal cycling conditions were as follows: 95 °C for 10 min, followed by 40 cycles of 95 °C for 15 s and 58 °C for 1min. The relative expression of mRNA in each sample was calculated by 2^−∆∆*C*t^ method.

### 4.9. Western Blot Analysis

The total protein of hypothalamus from each mouse was extracted using RIPA Lysis buffer (Biomiga, Santiago, CA, USA) and the BCA protein assay kit (Termo Fisher Scientific) was used to determine the protein concentration. The protein expressions of apelin and APJ were measured by western blot and the procedure was performed as previously described [56]. The 12% SDS-PAGE was selected to conduct transmembrane according to relative molecular weight of apelin and APJ, and then proteins were transferred onto polyvinylidene fluoride (PVDF) membranes. PBST with 5% nonfat milk was used to block the membranes, and the primary antibodies were apelin (M-77) (SC-33805, 1:100 dilution, Santa Cruz, Dallas, TX, USA), Apelin receptor Antibody (APJ) (PA5-21306, 1:500 dilution, Thermo Scientific) and β-Tubulin Monoclonal Antibody (A01030, 1:5000 dilution, Abbkine, Inc., Redlands, CA, USA). The SuperSignal^®^ West Pico Chemiluminescent Substrate (Thermo Scientific) was used to develop the membranes and the Tanon-5200 analysis system (Tanon, Shanghai, China) was used for exposure, then the optical density of protein bands were read by Tanon Gis software. 

### 4.10. Immunohistochemical Staining Analysis

The whole brains in 4% paraformaldehyde solution were used to prepare paraffin slices which included PVN and SON based on the mouse brain stereotaxic atlas [57]. The immunohistochemical stainingprocedure was performed as previously described [52]. In brief the steps were as follows: (1) the slices were dewaxed in graded xylene thrice each for 15 min; (2) dehydrated using graded ethanol thrice each for 1 min; (3) flushed with tap water for 3 min, then soaked in deionized water thrice each for 1 min; (4) heat induced epitope repair (HIER) was used for antigen retrieval; (5) washed the slices by PBST (0.01 M PBS with 0.1% Tween-20) thrice each for 5 min, and incubated with 3% H_2_O_2_ for 10 min, then washed again; (6) incubated with low lenthal serum for 60 min, then incubated with primary antibody (apelin: 1:200 dilution; APJ: 1:1000 dilution) at 4 °C overnight; (7) washed the slices by PBST thrice each for 5 min, then incubated with polymer helper for 30 min at room temperature and washed again; (8) incubated with the HRP conjugated anti-rabbit IgG polymer for 30 min at room temperature; (9) washed the slices by PBST thrice each for 5 min, then the DAB solution was used for coloration; (10) the slices were rinsed with tap water to stop the color reaction, then sealed with neutral gum after the counterstaining. 

A image analyzer (MIAS99, Fubo-Tech Co, Beijing, China) with a color video camera (TK-C1381, JVC, Beijing, China) and BX50 microscope (Olympus Co., Tokyo, Japan) was used to collect images of SON and PVN at high power magnification (400×), and the Imagepro Plus 6.0 software was used to obtain the average optical density (AOD) which is widely used in the image analysis [58,59].

### 4.11. Statistical Analysis

Data of the results were provided in the Supplementary Materials file, and the data were expressed as means ± standard errors of the means (SEM). SPSS 21.0 software was used to analyze the data, one-way analysis of variance (ANOVA) or non-parametric test was used for general data based on normality test and homogeneity test for variance, and least significant difference (LSD) method was adopted for the comparisons between groups. Besides, repeated measurement process of general linear model (GLM) was used to conduct one-way ANOVA analysis for repeated measured data (body weight and food intake), and multivariate analysis process of variance was used to make comparisons between groups on each time point (LSD method). GraphPad Prism 6.0 software was used to draw statistical graphs. A *p*-value < 0.05 was considered statistically significant.

## 5. Conclusions

A mouse model of CUMS was established successfully in this study, and Xiaoyaosan could significantly improve the general state and depressive-like behaviors of CUMS-induced mice. The apelin-APJ system is involved in the regulation of HPA axis, it also might be associated with the therapeutic mechanism of Xiaoyaosan. The study has proved that Xiaoyaosan could reverse the CUMS-induced changes of the apelin-APJ system by up-regulating the apelin level and down-regulating the APJ level in the hypothalamus. This would be an effective regulation point for the efficacy of Xiaoyaosan.

## Figures and Tables

**Table 1:** Primer sequences used in the qRT-PCR analysis.

Gene		Sequences
Apelin	forward	5′-CTGCTCTGGCTCTCCTTGAC-3′
	reverse	5′-CTCGAAGTTCTGGGCTTCAC-3′
APJ	forward	5′-CGCTCATTCCTGCCATCTAC-3′
	reverse	5′-AAGGTCAAGTCAGCCACTGC-3′

## Supplementary Materials

The following are available online.
