# Supplementary material for: Xiaoyaosan Improves Depressive-Like Behaviors in Mice through Regulating Apelin-APJ System in Hypothalamus
Source: Molecules. 2018 May 3;23(5):1073. doi: 10.3390/molecules23051073 (PMC6102542; doi:10.3390/molecules23051073)
Supplement: Supplementary file 1 [file molecules-23-01073-s001.pdf]

Supplementary Files

Xiaoyaosan Improves Depressive-Like Behaviors in Mice through Regulating Apelin-APJ System in Hypothalamus

Zhiyi Yan [1], Haiyan Jiao [1], Xiufang Ding [1], Qingyu Ma [2], Xiaojuan Li [1], Qiuxia Pan [1], Tingye Wang [1], Yajing Hou [1], Youming Jiang [1], Yueyun Liu [1] and Jiaxu Chen [1,*]

[1] School of Basic Medical Science, Beijing University of Chinese Medicine, Beijing 100029, China; 15010190928@163.com (Z.Y.); jiao.hy@foxmail.com (H.J.); d184728208@gmail.com (X.D.); 15652608965@163.com (X.L.); pqx1126@sina.com (Q.P.); Wty1307@163.com (T.W.); yajingHou@163.com (Y.H.); castenyy@gmail.com (Y.J.); chloelou@126.com (Y.L.)

[2] School of Traditional Chinese Medicine, Jinan University, Guangzhou 510632, Guangdong, China; 20140941026@bucm.edu.cn

* Correspondence: chenjiaxu@hotmail.com; Tel.: +86-10-6428-6656

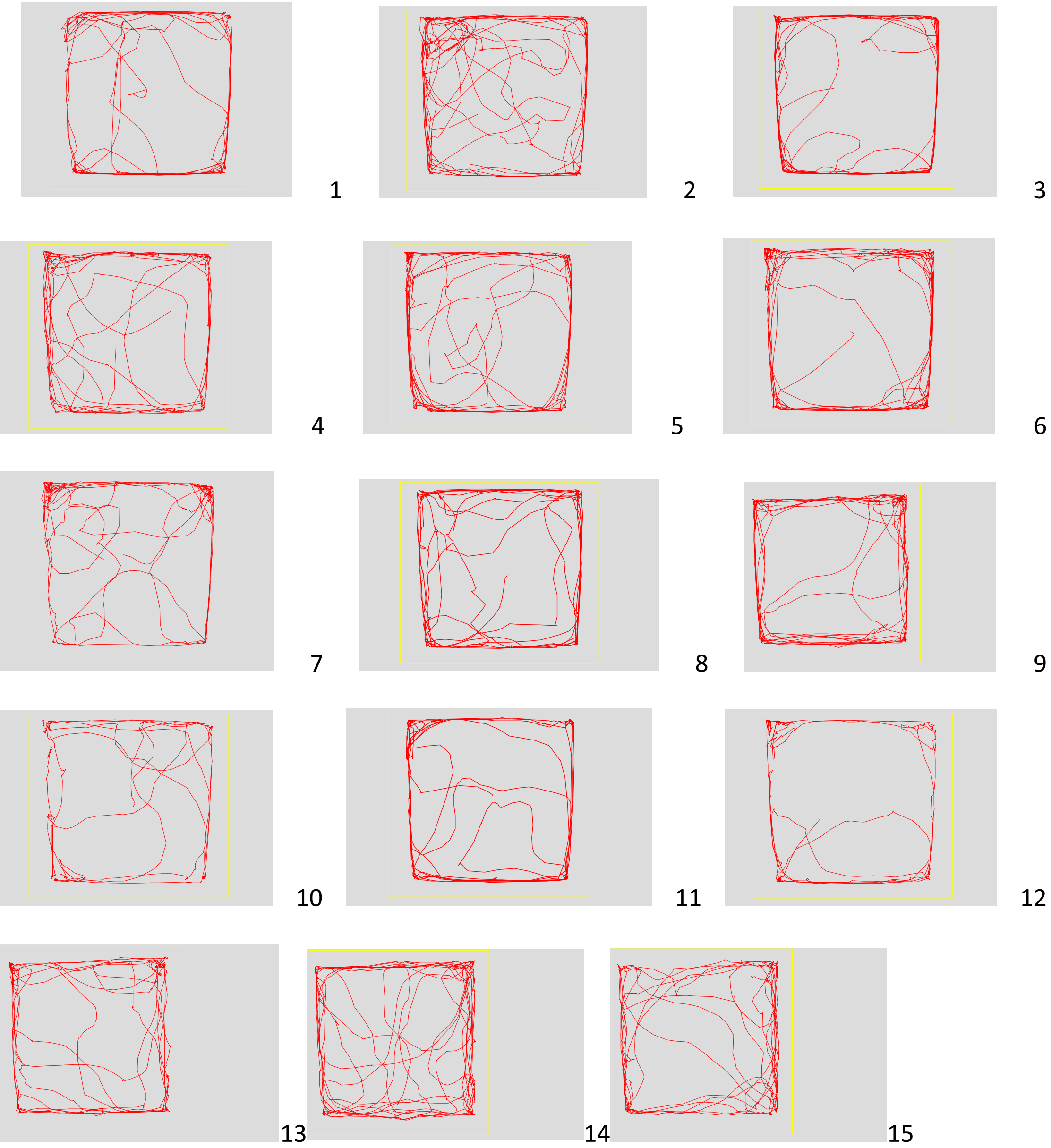

Figure S1. The open-field test moving trails of mice in control group at day 21.

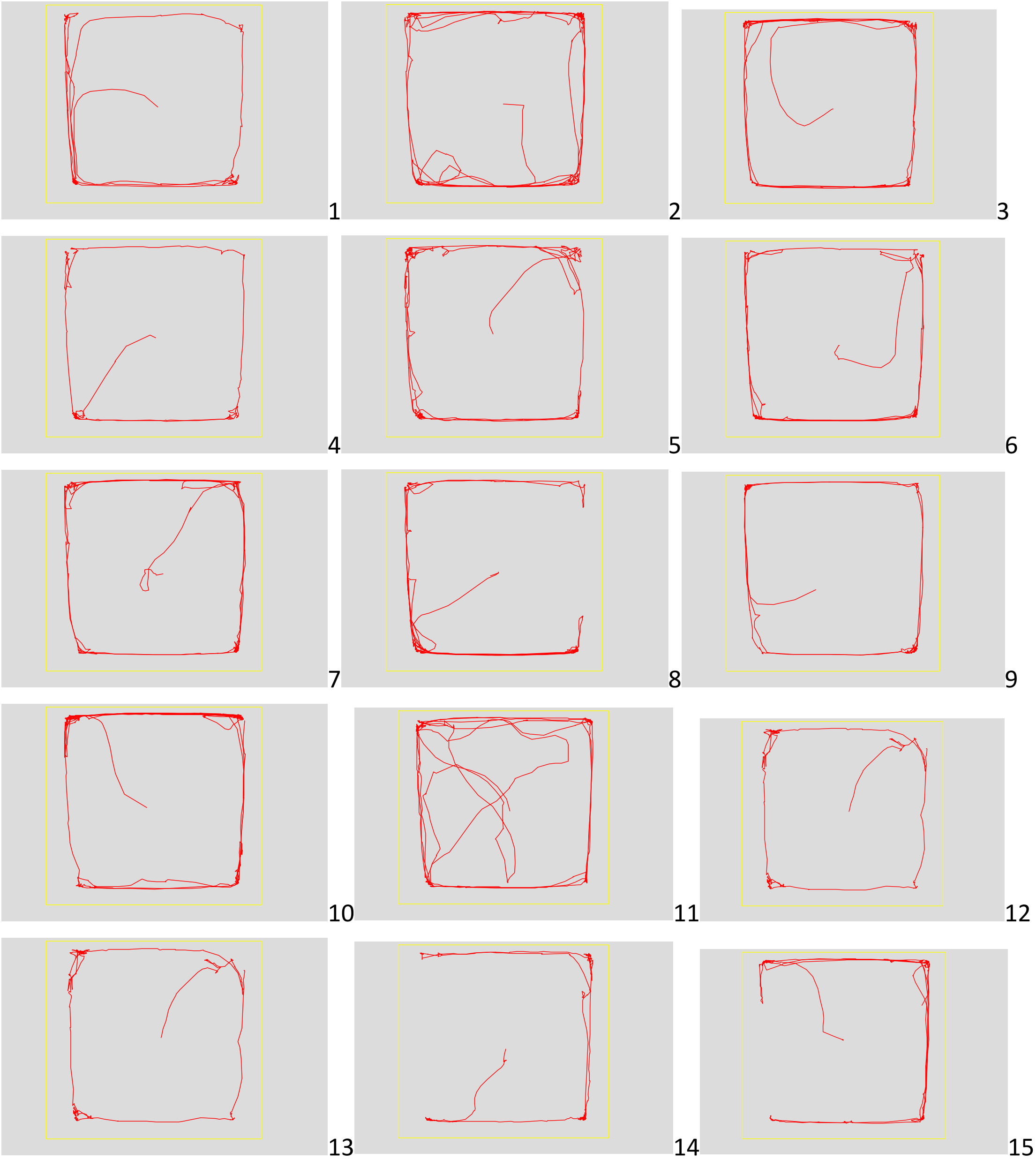

Figure S2. The open-field test moving trails of mice in model group at day 21.

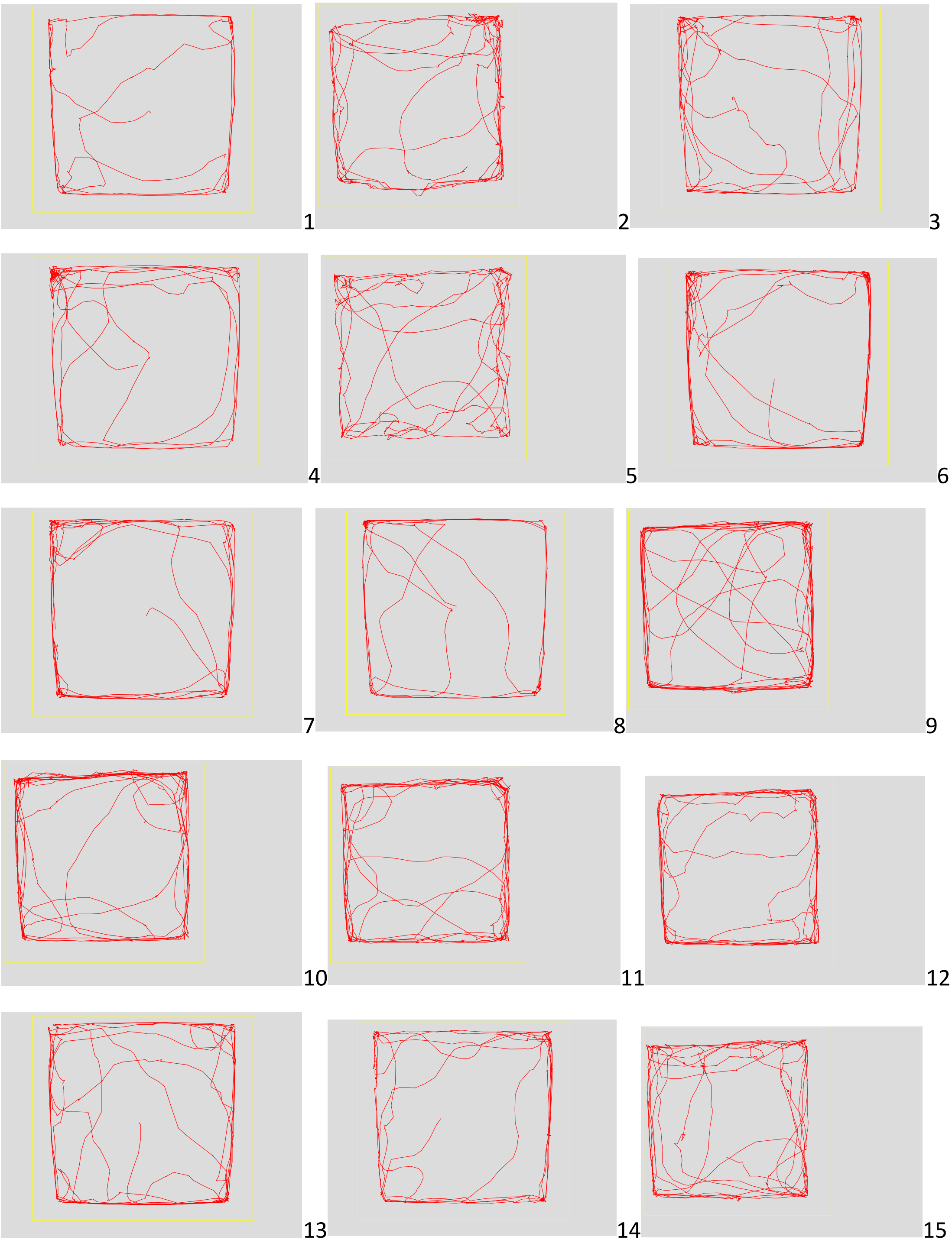

Figure S3. The open-field test moving trails of mice in Xiaoyaosan treatment group at day 21.

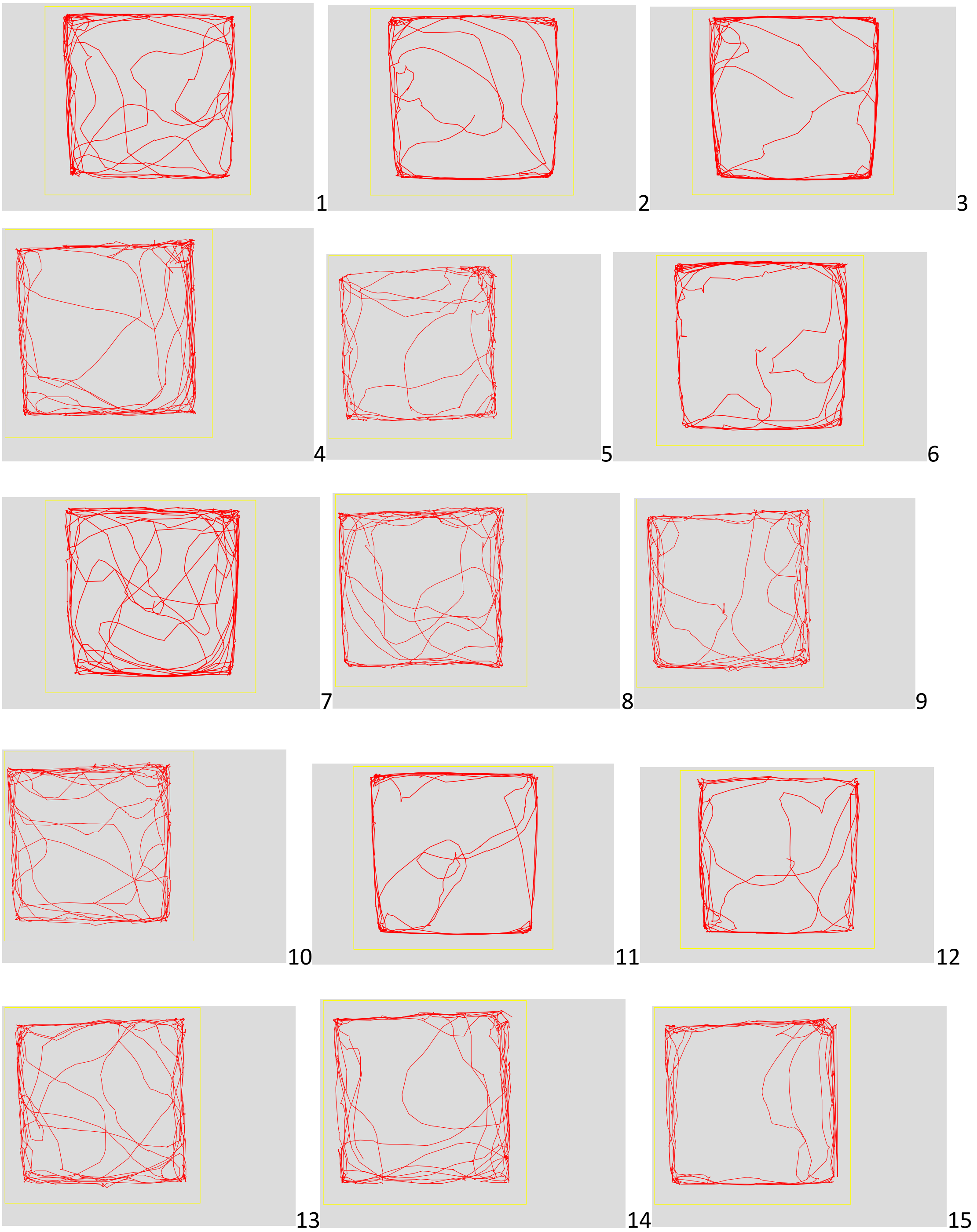

Figure S4. The open-field test moving trails of mice in fluoxetine treatment group at day 21.

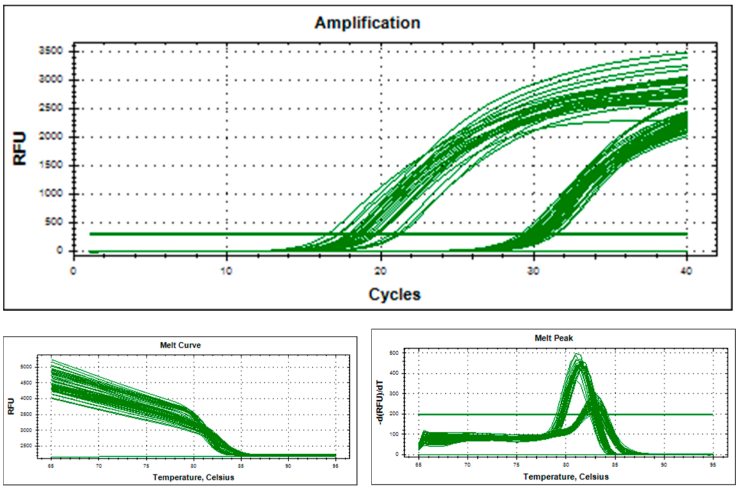

Figure S5. Apelin qRT-PCR Results of Model and Fluoxetine Treatment Groups

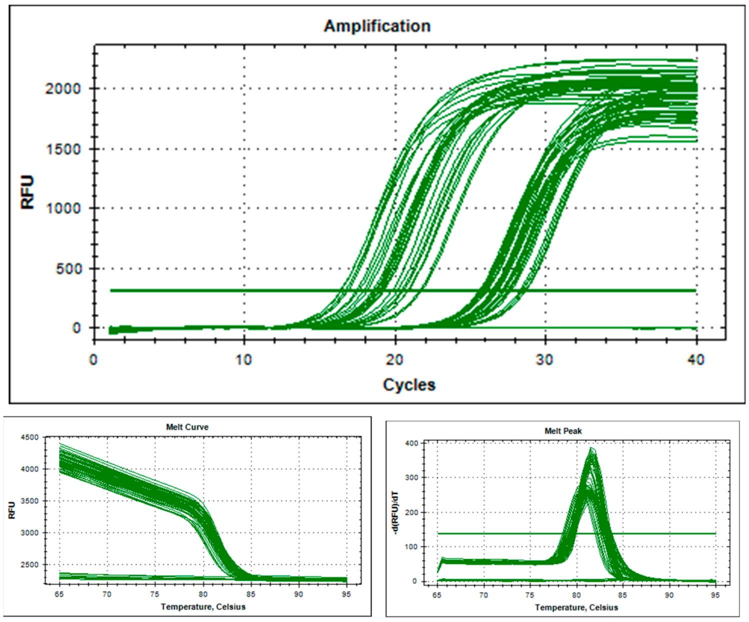

Figure S6. Apelin qRT-PCR Results of Control and Xiaoyaosan Treatment Groups

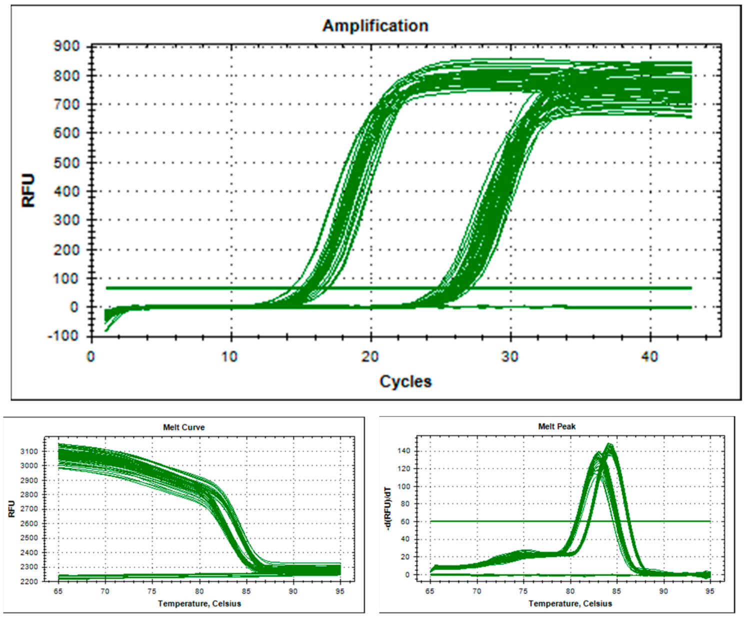

Figure S7. APJ qRT-PCR Results of Control and Fluoxetine Treatment Groups

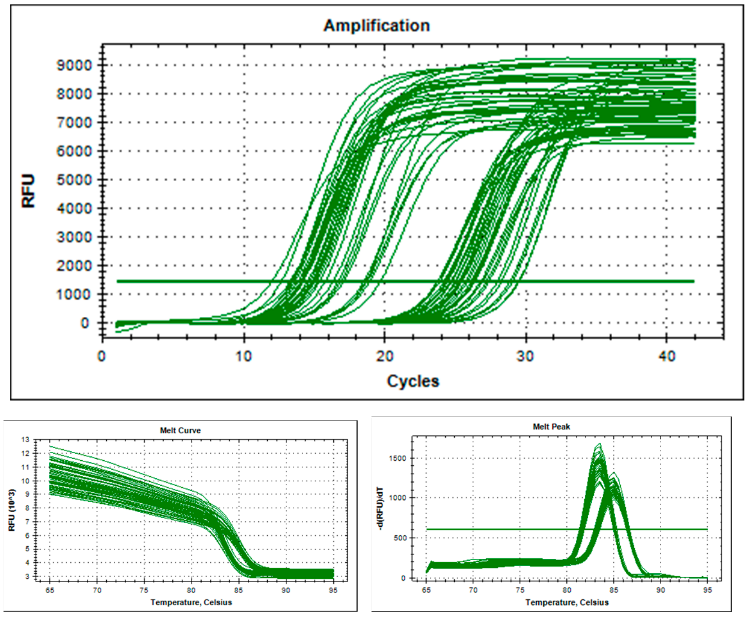

Figure S8. APJ qRT-PCR Results of Model and Xiaoyaosan Treatment Groups

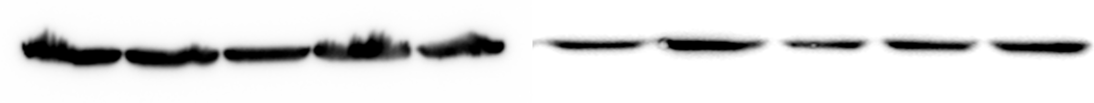

Control Group **Model Group**

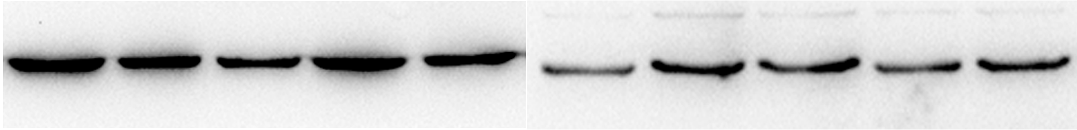

Xiaoyaosan Treatment Group **Fluoxetine Treatment Group**

Figure S9. Western blot Results of Apelin

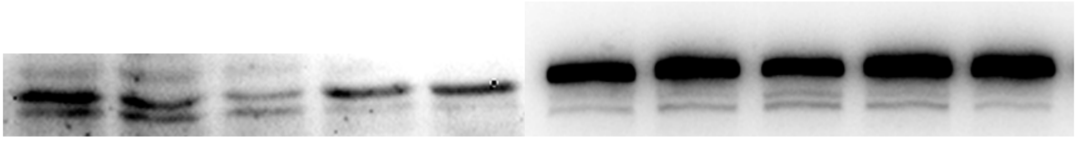

Control Group **Model Group**

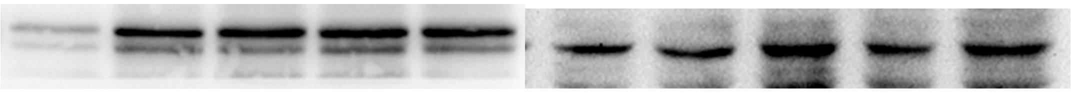

Xiaoyaosan Treatment Group **Fluoxetine Treatment Group**

Figure S10. Western blot Results of APJ

Supplement figure 11

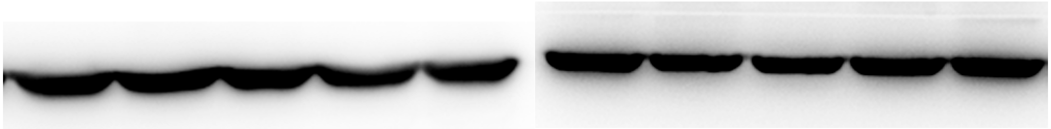

Control Group **Model Group**

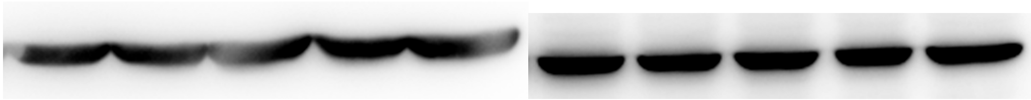

Xiaoyaosan Treatment Group **Fluoxetine Treatment Group**

Figure S11. Western blot results of β-tublin

Table S1. The total movement distance of mice in each group in the open-field test (cm)

Time	Day0					Day21			
Group	Control	Model	Xiaoyaosan	Fluoxetine		Control	Model	Xiaoyaosan	Fluoxetine
	2528.88	1968.59	2634.46	2521.82		3300.36	1278.48	1606.48	3133.02
	4176.98	2687.36	2781.27	3063.15		4352.82	1823.62	1967.04	2727.68
	2962.62	2928.8	2553.47	3157.91		4146.04	231.68	2290.6	3812.57
	2412.08	2726.83	3754.85	2930.92		3591.41	1405.85	2128.23	2306.29
	3146.29	3103.9	3328.82	2940.64		3957.09	1360.13	2938.49	2519.8
	1045.07	3151	3209.24	3641.05		3767	1541.1	2657.07	2840.02
	1867.69	2772.03	3612.71	3867.75		2678.87	1517.57	2492.5	4276.65
	4202.89	1479.49	3597.61	3421.34		1293.23	1144.51	1859.34	1068.08
	3092.03	3097.82	2476.51	2713.66		1960.12	1973.14	2598.91	1797.86
	2674.72	3939.67	3109.6	3123.08		1974.02	2043.5	2586.41	1679.31
	2928.2	3828.06	3637.54	2149		1451.92	790.22	2223.58	2691.92
	3133.71	3062.08	2578.74	3352.13		1802.75	2107.66	1521.67	2468.07
	3582.64	3937.02	3026.14	3126		1718.26	770.54	2930.53	1251.03
	3617.92	2611.01	2440.28	3006.58		1983.38	1301.79	2245.64	961.79
	3286.38	3579.39	2989.41	2839.65		1785.85	826.6	2222.54	2161.25

Table S2. The number of entries into central area of mice in each group in the open-field test (times)

Time	Day0					Day21			
Group	Control	Model	Xiaoyaosan	Fluoxetine		Control	Model	Xiaoyaosan	Fluoxetine
	8	14	5	6		6	1	4	5
	19	5	7	3		12	1	5	3
	7	1	5	4		4	1	8	2
	2	2	5	7		9	1	3	4
	5	4	6	3		6	1	3	7
	2	6	4	2		2	1	6	5
	2	10	8	10		8	3	3	4
	4	6	7	8		1	3	3	3
	2	3	5	7		1	2	4	3
	3	3	4	6		1	1	2	3
	3	1	7	2		5	1	2	9
	8	8	6	8		1	1	2	2
	2	4	3	5		1	1	5	3
	9	12	8	7		1	1	4	5
	4	7	5	3		3	3	2	2

Table S3. The central residence time of mice in each group in the open-field test (sec)

Time	Day0					Day21			
Group	Control	Model	Xiaoyaosan	Fluoxetine		Control	Model	Xiaoyaosan	Fluoxetine
	13.78	24.44	16.22	12		6.33	17.56	6.67	1.11
	41.11	8.22	7.78	6.67		23	13.11	2	1.78
	17.33	4.67	15.78	8		4.44	8	16	2.22
	7.78	14.44	4.89	5.33		4.44	3.56	3.78	4.89
	9.11	7.55	8.44	8.44		4.33	3.11	5.11	4
	3.56	14.67	16.89	14.22		3	8.89	4	3.56
	8.89	6.44	22	14.67		4.22	27.56	2.22	6.67
	9.33	7.55	18.89	11.11		4.11	5.55	4.89	2.67
	3.56	4.67	10.22	19.78		3.67	4.44	2	7.44
	2.67	2.22	2.44	8		1.44	5.33	2	1.33
	9.33	14.44	8.22	7.78		3.78	16.67	1.33	11.78
	9.11	24	8.89	13.11		2.67	10	0.67	2.44
	9.55	6.67	9.78	9.11		1.55	4.44	12.22	5.44
	7.33	12	7.55	6.67		1.55	4	5.55	3.55
	5.74	14.22	6.88	10.22		3.44	2.67	4.22	6.44
